# Oxyfunctionalisation of anisole and its selected reaction products by unspecific peroxygenases

**DOI:** 10.1016/j.bbrep.2025.102088

**Published:** 2025-06-10

**Authors:** Essi Rytkönen, Janne Jänis, Anu Koivula, Juha Rouvinen

**Affiliations:** a Department of Chemistry, University of Eastern Finland, P.O. Box 111, FI-80101, Joensuu, Finland; b VTT Technical Research Centre of Finland Ltd, P.O. Box 1000, 02044-VTT, Espoo, Finland

**Keywords:** Unspecific peroxygenase, Oxyfunctionalisation, Phenol, Hydroxylation, Demethylation, Oligomerisation

## Abstract

Unspecific peroxygenases (UPOs) are fungal enzymes capable of oxidizing hundreds of different organic compounds using hydrogen peroxide as the sole co-substrate. Aromatic ethers are among the less-studied substrates for UPOs, even though they offer a potential route to valuable phenolic compounds, such as those derived from the depolymerized lignin as a feedstock. Consequently, the oxyfunctionalisation potential of a panel of 30 UPO enzymes, along with *Agrocybe aegerita* UPO, was investigated using a simple aromatic ether, anisole. Anisole oxyfunctionalisation by the studied UPOs involved up to four consecutive reactions, resulting in the detection of 14 different products. Aromatic hydroxylation was the primary reaction pathway, leading to methoxyphenols or methoxybenzenediols. *O*-Demethylation also occurred, as phenol and benzenediols were identified among the products. As expected, different UPOs produced varying products and yields; however, guaiacol and 4-methoxyphenol were detected with every enzyme. The effect of ascorbic acid as a radical scavenger was also assessed, given that radical reactions leading to oligomerisation can occur with phenolic products. Generally, the presence of ascorbic acid broadened the product range, potentially due to the detection of trace products and a reduction in quinone formation. Owing to the broad product scope with anisole, biotransformations were also carried out with the identified phenolic intermediates to evaluate further oxidation routes. Based on these findings, a reaction pathway for anisole was proposed, offering insights into potential applications of UPOs in the tailored oxyfunctionalisation of phenolic substrates.

## Highlights

• Anisole and its first oxidation products were oxidised by unspecific peroxygenases.
• Anisole oxidation led to various products through demethylation and hydroxylation.
• Use of ascorbic acid as a radical scavenger led to the wider product scope.
• The specific reaction pathways for different products were proposed.

## 1 Introduction

Unspecific peroxygenases (UPOs, EC 1.11.2.1) are heme-containing fungal enzymes capable of one- and two-electron oxidations of a wide range of substrates, including aliphatic and aromatic compounds [1,2]. UPOs were first discovered from the black poplar mushroom (*Agrocybe* or *Cyclocybe aegerita*) some twenty years ago [3,4]. Since then, several other UPOs have been identified and characterised, e.g. those from an agaric fungus *Marasmius rotula*, *Coprinopsis cinerea* and *Myceliophthora thermophila* [[5], [6], [7], [8]]. UPOs have been considered as a missing link between cytochrome P450 monooxygenases and haloperoxidases, requiring only hydrogen peroxide to carry out oxidative reactions [1,2]. They can be divided into long and short UPO classes based on the length of their polypeptide chains [2,9]. Long and short UPOs share a similar overall 3D structure, but the active site pocket architecture differs. Long UPOs, such as the *A. aegerita* UPO (*Aae*UPO), are approximately 44 kDa proteins. They have a long C-terminal extension, which consists of two α-helices which partially limit the access to the active site. These UPOs are predominantly monomeric, with an arginine residue functioning as part of an acid-base pair alongside a conserved glutamate residue near the heme cofactor [1,10,11]. In long UPOs, a narrow channel lined with aromatic residues leads to the active site, favouring oxidation of smaller aromatic substrates [1,4,12]. The average mass of short UPOs is 29 kDa, and they are mainly dimeric proteins with a histidine residue as a charge stabiliser [1,9]. For example, *Hsp*UPO from the filamentous fungus *Hypoxylon* sp. EC38 can be classified into this class [13]. Short UPOs are capable of oxidizing larger substrates owing to their broader active sites, which predominantly contain aliphatic amino acid residues; nevertheless, their activity toward aromatic ring oxidation remains limited [1,4,11]. Both classes of UPO enzymes share many structural features, including extensive glycosylation and a heme prosthetic group, which serves as the catalytic cofactor and is coordinated by a cysteine residue within the conserved PCP motif [11,13,14]. Due to their versatility, UPOs have gained considerable attention recently for oxyfunctionalisation of C–H bonds in various organic compounds [12,13].

The natural function of UPOs is unknown, but it has been expected to be related to e.g. detoxification of organic compounds, as well as to biodegradation of compounds derived from lignin and other plant sources [1,10,15,16]. Lignin is a naturally abundant biopolymer that consists of three structural units; *p*-hydroxyphenyl, guaiacyl, and syringyl units [17]. These units are connected mainly by aryl ether and carbon-carbon bonds, and hydroxyl groups may also be substituted by different alkyl groups [18]. Due to its complex chemical structure, lignin degradation is challenging, but it can be depolymerized to low-mass oligomers and/or monomers by various techniques such as hydrothermal liquefaction or reductive catalytic fractionation [2,19]. Oxyfunctionalisation of aromatic compounds derived from lignin would offer versatile possibilities, e.g., for synthesis of agrochemicals and polymers [2]. *O*-Demethylation would also further convert the obtained ether compounds to more valuable phenols [20]. Thus, efficient demethylation and oxidation strategies would be beneficial in lignin valorisation, since lignin is a renewable source of carbon and majorly available, for example, as a pulp and paper industry side stream [18,19].

Fungi and their enzymes are of interest in lignin degradation and valorisation, since they can provide high turnovers in a sustainable manner [18,21]. Several enzymes, including P450 monooxygenases and UPOs, have attracted interest for their roles in dealkylation and aromatic hydroxylation, the key reactions for valorising depolymerized lignin [2,15,22,23]. Especially UPOs have been recognised to dealkylate various ether compounds and monolignols, and they have even been proposed to take part in the lignin degradation through demethylation [2,15,21,24]. Even beyond lignin valorisation, *O*-demethylation of ethers would be interesting, since it results in phenols that are precursors for other useful compounds, such as diols and other pharmaceutical precursors [22,25]. UPOs are also efficient enzymes in the hydroxylation of aromatic compounds, providing another route to phenols [25]. However, oxyfunctionalisation of aromatic ethers and phenols has not been widely studied with UPOs, although it would lead to promising applications.

The aim of this study was to characterise the oxyfunctionalisation potential of a panel of 30 UPOs towards a simple aromatic ether, anisole. The most studied UPO, *Agrocybe aegerita Aae*UPO, was used as a reference enzyme. The product scope was assessed, and their distributions were calculated to evaluate the selectivity of the UPOs towards demethylation and aromatic hydroxylation. As anisole oxyfunctionalisation was found to produce a large scope of products, biotransformations were also carried out with selected reaction products, using *Aae*UPO as the enzyme. Through these reactions, the oxidation potential towards phenolics could be evaluated, in addition to studying the possible routes to compounds observed in the anisole reactions. Based on the results, an oxidation pathway was proposed for consequent reactions observed. Finally, the effect of ascorbic acid as a radical scavenger on the reaction products was also evaluated, as it has previously been observed to affect the reaction outcome.

## 2 Experimental

### 2.1 Materials

The panel of unspecific peroxygenases used in the study was purchased from Aminoverse (Nuth, the Netherlands), which included 24 recombinantly produced UPOs and additionally 6 variants of one of the enzymes. The *Aae*UPO was expressed in a fungal host [26], and the enzyme was used as a non-purified culture supernatant. The substrates were obtained from Merck (anisole), Sigma Aldrich (phenol, guaiacol, 4-methoxyphenol, catechol, hydroquinone, resorcinol), TCI Europe (3-methoxyphenol) and BLD Pharmatech Ltd (2-methoxyhydroquinone). Besides previous compounds, following standards were used for product identifications: 1,4-benzoquinone (Sigma Aldrich), 2-methoxybenzoquinone (BLD Pharmatech Ltd.), 4-methoxyresorcinol (BLD Pharmatech Ltd.), 1,2,3-benzenetriol (Sigma Aldrich) and 1,2,4-benzenetriol (Sigma Aldrich). The substrates and standards were dissolved in acetonitrile (VWR Chemicals). Hydrogen peroxide solutions used in reactions were prepared from 35 % H_2_O_2_ from Merck, and the radical scavenger solutions from ascorbic acid, obtained from Honeywell Riedel-de-Haën. Ammonium acetate (Honeywell) dissolved in HPLC water (VWR Chemicals) was used as a buffer, and HPLC grade ethyl acetate (VWR Chemicals) was used for extraction and sample preparation. The samples were dried with Na_2_SO_4_ from Merck.

#### 2.1.1 Biotransformations with UPOs

The reactions with UPOs were carried out in 2 mL reaction tubes. The reaction mixtures contained UPO solution in 100 mM ammonium acetate (pH 7.5) and 4 mM of substrate dissolved in acetonitrile, leading to approximately 2 % (v/v) final concentration of acetonitrile. The total volume was 300 μL. Hydrogen peroxide solution was added in a single portion (6 μL, 200 mM) to the mixture in the beginning of the reaction, and again in the same volume after 2 h of reaction to reach the final concentration of 7.8 mM. The reactions containing a radical scavenger to reduce the peroxidase activity included additional ascorbic acid (10 mM). The reaction mixtures were incubated for 4 h at 30 °C in a benchtop shaker incubator (Thermomixer comfort, Eppendorf, Hamburg, Germany) with a rotation speed of 500 rpm. To stop the reactions, 300 μL of ethyl acetate was added and the mixtures were extracted for 30 min at 500 rpm, after which the organic phase was separated and further dried with Na_2_SO_4_. All the reactions were performed in duplicate.

To ensure that the substrates were not oxidised solely by hydrogen peroxide, negative controls were also performed. These reactions were performed as the UPO reactions but using ammonium acetate without the added enzyme. These negative controls for anisole included a reaction mixture containing 1) substrate and peroxide, 2) substrate, peroxide and ascorbic acid, 3) substrate and ascorbic acid, or 4) substrate only. The controls 1, 3 or 4 did not result in notable oxidation of anisole, whereas traces of guaiacol and 4-methoxyphenol were observed in the EIC *m/z* 110 for control 2. Additionally, the negative controls 1 and 2 were performed for the phenolic products. Also, experiments with *Aae*UPO in the presence of ascorbic acid but absence of H_2_O_2_ produced only minor amounts of guaiacol and 4-methoxyphenol detected in the EIC. Recently, Deng et al. have reported greater oxidation of substrates with ascorbic acid than with hydrogen peroxide [27].

#### 2.1.2 *GC*–*FID*/*MS analysis*

The obtained extracts were analysed by a Bruker/Scion 456-GC gas chromatograph (equipped with a PAL-RSI autosampler) connected to a Bruker timsTOF mass spectrometer (Bruker Daltonics GmbH, Bremen, Germany), using atmospheric pressure chemical ionization (APCI). The system was also equipped with an auxiliary flame ionization (FID) detector. The obtained extracts were diluted 2:3 (v/v) with HPLC grade ethyl acetate. The GC-FID/MS analysis was conducted with a Restek RXi-5Sil MS capillary column (30 m × 0.25 mm i.d., 0.25 μm film thickness). The analysis was performed as follows. The injector was held at 280 °C, and a split injection with a volume of 1 μL and a split ratio of 10:1 was used. The carrier gas was helium at a flow rate of 1.0 mL/min. The temperature ramp was the following: 40 °C (2 min hold), then 10 °C/min to 180 °C (2 min hold). The transfer line between GC and MS was kept at 285 °C. For MS, the ion source was operated in the positive ion mode with the following settings: capillary voltage 3000 V, corona needle 3000 nA, vaporizer temperature 200 °C, nitrogen gas flow 2.5 L/min, drying gas temperature 220 °C, and *m/z* range 50–800. Bruker Compass HyStar 6.0 and otofControl 6.2 software were used for the instrument control and data acquisition, and the data post-processing and analysis was carried out with Bruker Compass DataAnalysis (version 5.1).

#### 2.1.3 Product identifications

Potential oxyfunctionalisation products were identified by comparing them with authentic standards or the published data. The retention times and mass spectra of the product standards were compared to the data obtained from the UPO reactions. Otherwise, the identifications were based on the retention index data (obtained from Pubchem [28], using a semi-standard nonpolar column) and the mass spectra (NIST WebBook [29]), published previously. The retention indices were calculated as the linear retention indices [30] by comparing the retention times to the *n*-alkane standard (C_8_–C_20_; Sigma Aldrich), measured separately using the identical analysis program. When necessary, extracted ion chromatograms (EICs) were also calculated for selected products to enhance their detection and identification.

## 3 Results and discussion

### 3.1 Anisole

**Fig. 1:**
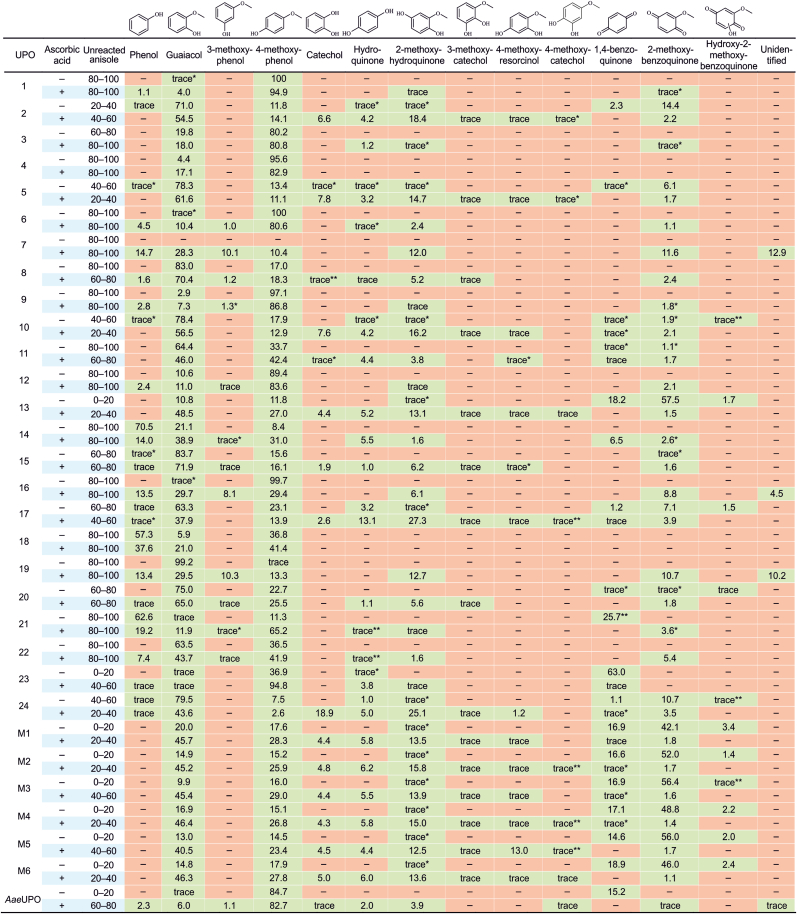
Anisole oxyfunctionalisation products with UPO enzymes and their distribution. The relative product abundances (%) have been calculated based on the FID areas and are average values from duplicate reactions. When a certain product was not observed with FID but only based on MS signal (EIC), or its relative abundance was below 1 %, it has been marked as “trace”. ∗ = product was observed only in the first screen, ∗∗ = product was observed only in the second screen. The amount of unreacted anisole (%) was calculated by comparison to negative control reactions.

Anisole is a simple aromatic ether that either demethylates or undergoes aromatic hydroxylation by the UPO activity. Both reaction types were observed for anisole, which led to observation of 14 distinct products across the studied UPO enzymes (Fig. 1). The exemplary FID chromatograms and the mass spectra for the UPO reactions are provided as Supplementary Information (Figs. S1–S4). One of the products remained unidentified, as the obtained data did not match with any possible compound. Aromatic hydroxylation was the prevalent route for anisole oxyfunctionalisation, leading to guaiacol and 4-methoxyphenol. 3-Methoxyphenol was also observed, but more rarely, indicating that all three positions from the methoxy substituent could be hydroxylated. In addition, dihydroxylation occurred during the reaction since 2-methoxyhydroquinone was detected. This product was further oxidised to 2-methoxybenzoquinone, which was hydroxylated even further with some of the enzymes. Additionally, dihydroxylated products, i.e., 4-methoxyresorcinol, 3-methoxycatechol and 4-methoxycatechol, were also observed, but mostly in trace amounts.

Although aromatic hydroxylation was the major reaction type observed, demethylation had also occurred since there were products that cannot be formed from anisole without the removal of the methoxy group. The demethylation product of anisole, phenol, was detected with about half of the UPOs. However, phenol is known to rapidly oxidize further or to form oligomers in absence of a radical scavenger [31], so it may only be transiently present in the reaction mixture. The combinations of aromatic hydroxylation and demethylation were also observed, since catechol and hydroquinone were identified as products. To form diols from methoxyphenols, demethylation should occur after aromatic hydroxylation. However, diols could also be formed through phenol oxidation, where anisole demethylation is followed by aromatic oxidation [[31], [32], [33]]. Nevertheless, anisole can be demethylated with UPOs, but this route is yet to be determined.

Overall, anisole reacted consecutively four times to obtain hydroxy-2-methoxybenzoquinone as the final product. Three of these oxidations were aromatic hydroxylations, which demonstrates the ability of UPOs to efficiently oxidize phenyl rings. Nine of the identified products resulted from hydroxylation reactions, accounting for up to 95 % of the products across the whole UPO panel. Demethylation potential of UPOs was to lesser extent, since only three of the products were formed from routes involving dealkylation. In average with all UPOs, under 5 % of the products formed through demethylation. There were some exceptions, such as UPOs 14, 18, 21, for which the demethylation products constituted around 60 % of total products without ascorbic acid, and 20 % with ascorbic acid. Also, the variant UPOs M1–M6 and their parent UPO 13 resulted in higher abundances of demethylation products.

As expected, there were variations between the reaction outcomes for different UPOs. Guaiacol and 4-methoxyphenol were produced by all studied UPOs, although the preferences towards *o*- and *p*-hydroxylations were different. From the studied UPOs, 20 UPOs preferred *o*-hydroxylation and 11 UPOs *p*-hydroxylation based on the product distributions. Methoxyphenols were the major products in most cases, since they are the result of the first oxidation of anisole. However, for example, with UPOs 17 and 24, further reactions were rather prominent in the presence of ascorbic acid. The six variant enzymes and their parent UPO also transformed anisole efficiently to the overoxidized product, 2-methoxybenzoquinone, without a radical scavenger, as its relative abundance was around 50 %. Even with ascorbic acid, the second aromatic hydroxylation to 2-methoxyhydroquinone was considerable. Furthermore, the variant UPOs essentially led to the same products with similar relative abundances.

The effect of ascorbic acid as a radical scavenger was also evaluated, as it has been said to inhibit the peroxidase activity of UPOs, thus preventing polymerisation reactions and concomitant quinone formation [1,14,25]. Generally, anisole reactions including ascorbic acid led to a wider range of products (Fig. 1), indicating that polymerisation reactions could be rather prominent in the absence of a radical scavenger. For most UPOs, only guaiacol, 4-methoxyphenol and 2-methoxybenzoquinone were observed without ascorbic acid in notable amounts. These compounds could potentially form phenoxyl radicals and undergo oligomerisation through the peroxidase activity of UPOs [12], although no coupling products were detected. However, the absence of oligomeric products could be due to analysis conditions as well. For example, the temperature program was only up to 180 °C, whereas oligomers might require higher temperatures to be detected by GC. It is also possible that they were not efficiently extracted from the reaction mixture and should have been analysed by another method such as liquid chromatography.

A wider range of products with a radical scavenger can be partially explained by quinone formation. For instance, 2-methoxyhydroquinone was present in notable amounts only in the presence of ascorbic acid. Hydroquinone was also observed with ascorbic acid, but typically not without it. It has been observed that the presence of a radical scavenger prevents the formation of quinones from hydroquinones [31,34]. However, ascorbic acid did not completely inhibit quinone formation, as 2-methoxybenzoquinone was observed with many UPOs even in the presence of it, although in low abundance. Traces of 1,4-benzoquinone were also observed for some of the UPO variants even with the radical scavenger. Also, many of the additional products were present in trace amounts, such as methoxybenzenediols. These might not form if the oligomerisation reaction is the dominant one.

Anisole oxyfunctionalisation reactions have not been studied with UPOs before, and the previous studies have mostly concerned cytochrome P450 monooxygenases. For example, a rather selective anisole conversion to guaiacol with only minor amounts of 4-methoxyphenol was reported by P450 BM3 monooxygenase and its variants [35]. However, *meta*-hydroxylation was not observed [35], contrary to the findings with the UPO panel enzymes in this work. The P450 BM3 systems with dual-functional small molecules (DFSMs) have also been developed to guide anisole transformation towards demethylation [20,22]. Whereas the wild-type enzyme system provided only small quantities of guaiacol and 4-methoxyphenol via aromatic hydroxylation, the double-point mutants could achieve 100 % selectivity towards *O*-demethylation [22]. Although the same products were identified with UPOs, it seems that UPOs are more versatile enzymes than P450s, as they transformed anisole also to 3-methoxyphenol, and further oxidised the formed products. A cleavage of other ethers has been studied previously with *Aae*UPO [24], suggesting that the observed anisole demethylation is reasonable. Additionally, the studies with lignin dimers and monolignols have revealed that UPOs are able to demethylate and hydroxylate compounds with ether bonds [2,21,24].

### 3.2 Biotransformations with selected reaction products

**Fig. 2:**
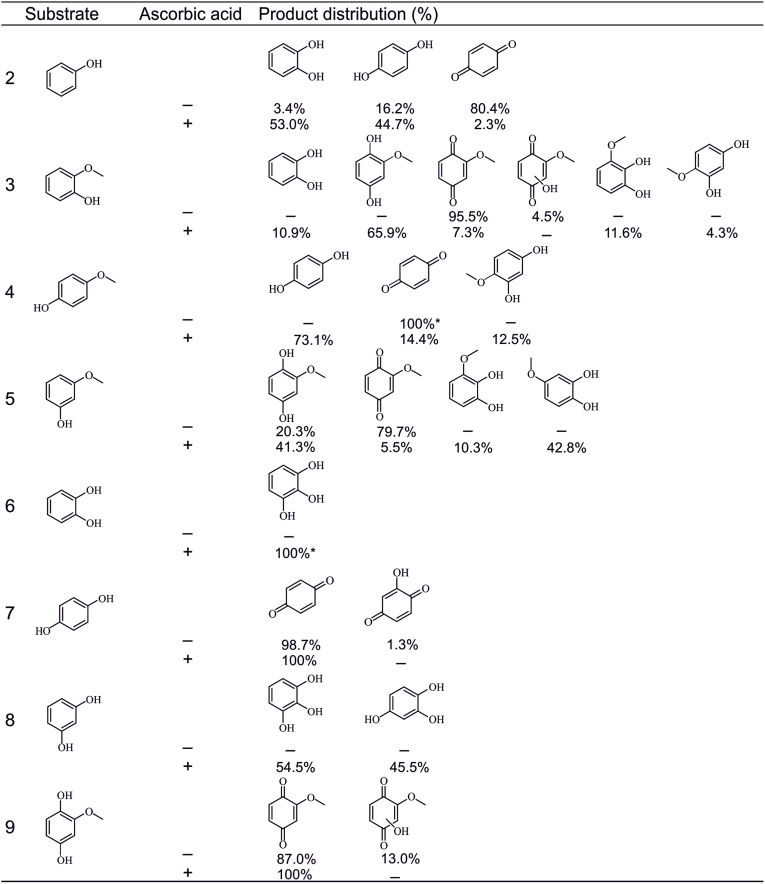
Observed products in the oxidation of eight phenolic compounds with *Aae*UPO and their relative abundances. The product distributions (%) are based on the FID responses and are average values from duplicate experiments. ∗ = product was observed only in trace amounts based on the EIC.

The product range for anisole was broad. Some observed products did not form directly from anisole but through subsequent oxidation reactions. To clarify the reaction pathways leading to specific products, such as 2-methoxybenzoquinone, additional reactions were conducted using eight phenolic compounds identified as reaction products: phenol, guaiacol, 3-methoxyphenol, 4-methoxyphenol, catechol, resorcinol, hydroquinone, and 2-methoxyhydroquinone. The most studied UPO, *Aae*UPO, was chosen as the enzyme because it yielded a broad product scope for anisole. Aromatic hydroxylation and quinone formation were identified as the primary pathways for increasing the oxidation state of the phenolic products. Fig. 2 illustrates the observed products and their distributions across the studied substrates.

Phenol is a possible reaction product through demethylation of anisole, although it has been observed to react further quite rapidly [31]. When phenol was oxidised with *Aae*UPO, catechol and hydroquinone were observed as products. Additionally, in the absence of ascorbic acid, 1,4-benzoquinone was detected as the dominant product. Over 95 % of the products formed through *p*-hydroxylation, whereas in the presence of ascorbic acid, a ratio of *o*- and *p*-hydroxylation products was almost 1:1. Previously, *Cma*UPO from *Coprinopsis marcescibilis* and r*Mfi*UPO from *Marasmius fiardii* PR-910 also transformed phenol to hydroquinone and catechol. For *Cma*UPO, the preference was towards the *o*-hydroxylation with 73 % of catechol, while r*Mfi*UPO preferred *p*-hydroxylation with 80 % of hydroquinone [32,33]. Phenol formed from benzene by *Aae*UPO was immediately hydroxylated to catechol, hydroquinone, and their respective benzoquinones with a preference towards *p*-hydroxylation [31], which is consistent with the findings in this study. Additionally, oligomerisation products were reported in the absence of ascorbic acid [31]. In another study, *Aae*UPO yielded *para*-oxidised products, in addition to other products which were not identified further [34]. The phenol oxidation by *Aspergillus brasiliensis* UPO (*Abr*UPO) also resulted in the formation of hydroquinone and benzoquinone [34]. A monolignol, 4-propylphenol, was also oxidised in its *ortho*-position by *Hsp*UPO, forming a 1,2-diol, analogous to catechol [2].

Guaiacol oxidation resulted in demethylation and aromatic hydroxylation along with a further oxidation of the products. In the presence of ascorbic acid, demethylation to catechol was observed along with a major hydroxylation to 2-methoxyhydroquinone. The hydroquinone was further oxidised to 2-methoxybenzoquinone. Additionally, 3-methoxycatechol and 4-methoxyresorcinol were identified as the aromatic hydroxylation products. In the absence of ascorbic acid, 2-methoxybenzoquinone was detected as the major product. A peak in the GC chromatogram, corresponding to the molecular formula of C_7_H_6_O_4_, was also observed. This was proposed to be the hydroxylated form of 2-methoxy-1,4-benzoquinone (C_7_H_6_O_3_). The previous results of guaiacol with P450 monooxygenases also indicated both demethylation and aromatic hydroxylation reactions [22]. With the P450 BM3-DFSM system and its different variations, guaiacol was oxidised to 2-methoxyhydroquinone, 4-methoxyresorcinol, and 3-methoxycatechol. Demethylation to catechol was also observed, and the selectivity towards catechol could be improved by mutations [20,22]. Another P450 enzyme from the CYP255 family was also able to transform guaiacol into catechol [23]. Concerning the related reports with UPOs, 4-propylguaiacol underwent demethylation and aromatic oxidation with *Hsp*UPO, resulting in 1,2-diol and 3-methoxy-5-propylbenzene-1,2-diol, respectively [2]. This result is consistent with our present findings using AaeUPO and guaiacol. With *Cma*UPO, 4-propylguaiacol was mostly dimerised, although the activity was reduced in the presence of ascorbic acid [2].

Oxyfunctionalisation of 3-methoxyphenol also led to the aromatic hydroxylation of the substrate. It was transformed to 2-methoxybenzoquinone and 3-methoxycatechol in the absence of ascorbic acid, and to 2-methoxyhydroquinone and 3- and 4-methoxycatechols in the presence of ascorbic acid. The products indicate aromatic hydroxylation in the *ortho*- and *para*-positions (in relation to the phenolic hydroxyl group). There is no previous data on the 3-methoxyphenol oxidation by peroxygenases for comparison, but based on the guaiacol oxidation patterns, these results seem plausible. However, 4-methoxyphenol oxidation seemed less prominent. Only small quantities of hydroquinone, 1,4-benzoquinone, and 4-methoxycatechol were observed in the presence of ascorbic acid. Without the radical scavenger, only traces of 1,4-benzoquinone were observed based on the EIC. However, the reaction mixture turned yellow during the incubation, which could possibly indicate oligomerisation of the substrate. Polymerized quinones strongly absorb in the blue region of the visible spectrum (450–500 nm), giving solutions a yellow to orange appearance. A weak signal observed for the substrate also supports the prominent oligomerisation reactions, even though these products were not detected with GC. This is probably because they were not extracted from the reaction mixture, since the colour of the solution was retained after the extraction.

Catechol oxidation without a radical scavenger seemed to yield no products, or they were not detected. Still, the solution turned dark brown during the reaction, indicating that some product(s) had been formed. 1,2-Benzoquinone could be a possible product since hydroquinones also oxidised to corresponding quinones. However, oligomerisation products are more plausible, since ascorbic acid was not present, and the substrate was not detected. The coloured product was not efficiently extracted by ethyl acetate (the colour remained brown), which would indicate a rather high degree of oligomerisation. Also, a similar, brown-coloured product was previously identified as a polycatechol in the oxidation of catechol by a thermophilic peroxidase from *Myceliophthora thermophila* [36], supporting oligomerisation. In the presence of ascorbic acid, only traces of 1,2,3-benzetriol were formed via aromatic hydroxylation but no other products were observed. Similarly, in the study with *Aae*UPO and *Abr*UPO, no products were reported in the catechol oxidation by either enzyme [34]. However, with *Mro*UPO of *Marasmius rotula*, 1,2-benzoquinone was reported as the product, like with *Aae*UPO in the consequent benzene oxidations [31,37]. Additionally, traces of 1,2,4-benzenetriol via further aromatic hydroxylations could be observed with *Aae*UPO [31]. Thus, the results of catechol oxidation slightly differ from the previous reports, which could be related to the trace levels of products formed.

Hydroquinone was transformed into 1,4-benzoquinone both in the presence and absence of ascorbic acid. In addition, a product with the molecular formula of C_6_H_4_O_3_ was formed without ascorbic acid, and the same compound was identified with 1,4-benzoquinone as the substrate. The product can most probably be identified as 2-hydroxy-1,4-benzoquinone, even though the retention index for the compound is not available. Similarly, hydroquinone oxidation yielded 1,4-benzoquinone with *Mro*UPO along with other coupling products as side products [37]. The earlier study with *Aae*UPO and *Abr*UPO also reported that differently substituted hydroquinones efficiently transform to 1,4-benzoquinones [34]. Especially in the absence of a radical scavenger, oxidation to quinone was rather complete, whereas it decreased with an increasing radical scavenger concentration [34]. A similar effect was observed in the present study; in the GC-FID analysis, 92 % of the total peak area was attributed to 1,4-benzoquinone without ascorbic acid, whereas this value dropped to only 2 % in the presence of ascorbic acid. In addition to benzoquinone, *Aae*UPO has been able to further hydroxylate both hydroquinone and benzoquinone, leading to traces of 2-hydroxy-1,4-benzoquinone and 1,2,4-benzenetriol [31], which was not observed in this work. The negative controls also led to benzoquinone formation, but with notably lower conversion rate, so hydroquinone conversion may not be fully attributed to the enzyme activity.

Resorcinol was not detected as a product of anisole oxidation; however, it was evaluated as a substrate because it could theoretically form via the demethylation of 3-methoxyphenol. The UPO reactions transformed resorcinol into two triols, 1,2,3-benzenetriol and 1,2,4-benzenetriol. In the absence of ascorbic acid, these were present only in trace amounts, suggesting that further oligomerisation occurred as the primary reaction, even though it was not directly observed. Previously, resorcinol conversion has been rather efficient with *Aae*UPO when compared to *Abr*UPO, which barely converted resorcinol. However, the identity of the products was not disclosed, as the study focused on the formation of hydroquinones [34]. *Mro*UPO was found to oxidize resorcinol, though the reaction products were not discussed beyond noting that the polymerisation was efficient [37].

2-Methoxyhydroquinone was tested as a substrate because many UPO panel enzymes appeared to produce it from anisole. The reaction with *Aae*UPO produced mostly 2-methoxybenzoquinone, but a compound with a formula of C_7_H_6_O_4_ was also observed in absence of ascorbic acid. As proposed earlier, this is presumably 2-methoxybenzoquinone that has been hydroxylated either in the *ortho*- or *para*-position. However, the exact structure could not be verified due to the lack of authentic analytical standards or reference retention index data for these compounds. The product was also observed when 2-methoxybenzoquinone was used as a substrate, confirming further hydroxylation of the 2-methoxyhydroquinone oxidation product. In the presence of ascorbic acid, benzoquinone was still formed from 2-methoxyhydroquinone, but the quinone-substrate ratio was notably smaller. This is reasonable according to the previous findings about ascorbic acid, which have revealed that it reduces the quinone formation, instead of completely inhibiting it [31,34]. The hydroxylated benzoquinone was not detected, probably due to the decreased quinone formation. However, 2-methoxyhydroquinone was also oxidised to benzoquinone in the negative controls without the enzyme, although the conversion was lower. Therefore, the oxidation may not be completely due to enzymatic activity.

**Fig. 3:**
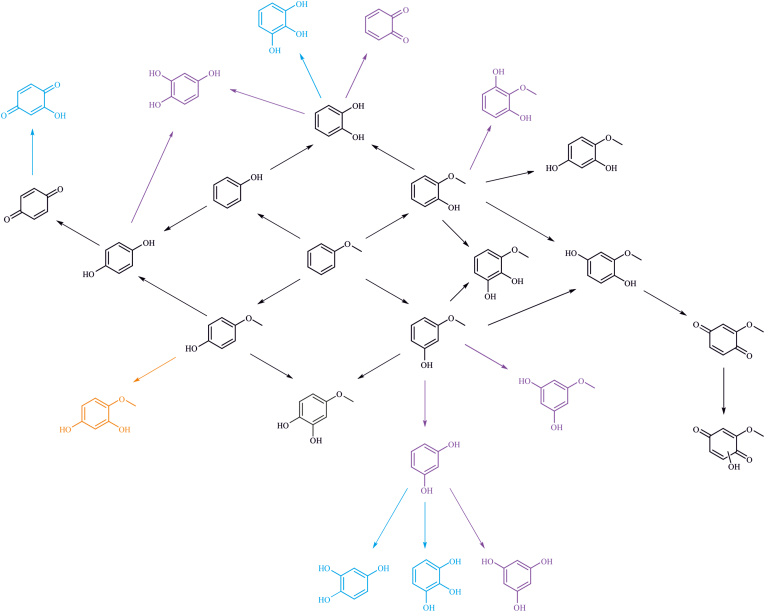
Proposed reaction routes for anisole oxyfunctionalisation. Based on these pathways, certain products (e.g. catechol) can form via different routes. The compounds coloured in purple are the theoretical products that were not observed in the anisole or phenol reactions. A blue colour signifies the products that were observed only in the reactions with oxidised intermediates (i.e. catechol, resorcinol, and 1,4-benzoquinone), but not in the anisole transformations. The product in orange (4-methoxyresorcinol) was not observed in the reaction with 4-methoxyphenol although it is theoretically possible, but it was identified from the anisole reactions.

The results with phenolic substrates confirm that all the observed products from anisole reactions are reasonable through overoxidation of the first-generation products, i.e., methoxyphenols and phenol. The consecutive oxidations probably occur since the oxidised products are more water-soluble and consequently better substrates for the enzyme. Oxidation of phenolic compounds was tested only with a single UPO, so it is possible that the product scope would be different with other UPOs due to the versatility of the enzymes and their catalytic sites. Nevertheless, based on the reactions with phenolic products, the reaction routes for anisole oxyfunctionalisation are proposed (Fig. 3). Depending on the UPO and its reactivity, some of the observed products, e.g. catechol, could form through different routes. It is also possible that several routes operate at the same time, leading to the same product, such as 2-methoxyhydroquinone or 2-methoxybenzoquinone, which were observed in notable amounts with most UPOs. Based on the findings of this study, anisole oxyfunctionalisation with UPOs often leads to overoxidation to various products via several routes. Overoxidation might be reduced with shorter reaction times or protein engineering, which could also allow selective production of a specific compound.

## 4 Conclusions

Anisole oxyfunctionalisation by UPO enzymes led to a wide array of products via demethylation and hydroxylation reactions occurring in the aromatic ring. Anisole was oxidised consecutively up to four times, leading to different product distributions according to the used enzyme. A total of 14 products were observed across the studied UPOs, including methoxyphenols, diols, and methoxybenzenediols. Aromatic hydroxylation was the preferred oxidation route for all the UPOs, resulting in guaiacol and 4-methoxyphenol formation. Nevertheless, demethylation occurred to the lesser extent, since phenol and benzenediols were observed. The presence of ascorbic acid as a radical scavenger, resulted in the wider product distributions, since both quinone and hydroquinone products could be observed. Ascorbic acid may reduce oligomerisation caused by UPO peroxidase activity, allowing trace products like various methoxybenzenediols to be detected. Thus, radical scavengers can be used to modulate the product distribution toward smaller products.

To discover the origin of the overoxidized products, biotransformations were also carried out with the identified products as substrates. The UPOs efficiently hydroxylated various phenolics with a reduced demethylation activity. A quinone formation was also the major pathway to the observed products. The effect of ascorbic acid was similar than with anisole, as it decreased the quinone formation and supposedly polymerisation, leading to the detection of more compounds. However, hydroxylated quinones were not detected in the presence of radical scavenger, probably because the formation of quinones was not as prominent. Based on the reactions with the product phenolics, possible pathways for anisole products could be proposed. Certain products, such as catechol, could be formed via different routes, or several routes could be present concomitantly, favouring certain products. Altogether, UPOs were capable of aromatic hydroxylation and demethylation of anisole and its primary oxidation products, which highlight their marked potential in lignin valorisation for manufacturing of important platform and/or specialty chemicals.

## CRediT authorship contribution statement

**Essi Rytkönen:** Writing – original draft, Validation, Methodology, Investigation, Formal analysis, Data curation. **Janne Jänis:** Writing – review & editing, Supervision, Project administration, Methodology, Conceptualization. **Anu Koivula:** Writing – review & editing, Validation, Resources, Funding acquisition, Conceptualization. **Juha Rouvinen:** Writing – review & editing, Supervision, Resources, Project administration, Funding acquisition, Conceptualization.

## Funding

This work was supported by the Research Council of Finland (grant number 355455 and 355456/Oxyfunc). The mass spectrometry facility is supported by Instruct Centre Finland (FINStruct) 10.13039/100030846Biocenter Kuopio, and the Research Council of Finland.

## Declaration of competing interest

The authors have nothing to declare.

## Data Availability

Data will be made available on request.
