# Supplementary material for: Oxyfunctionalisation of anisole and its selected reaction products by unspecific peroxygenases
Source: Biochem Biophys Rep. 2025 Jun 10;43:102088. doi: 10.1016/j.bbrep.2025.102088 (PMC12182302; doi:10.1016/j.bbrep.2025.102088)
Supplement: Multimedia component 1 [file mmc1.pdf]

Supplementary information

Oxyfunctionalisation of anisole and its selected reaction products by unspecific peroxygenases

Essi Rytkönen[a], Janne Jänis[a], Anu Koivula[b] and Juha Rouvinen[a]*

[a]Department of Chemistry, University of Eastern Finland, P.O. Box 111, FI-80101 Joensuu, Finland

[b]VTT Technical Research Centre of Finland Ltd, P.O. Box 1000, 02044-VTT, Espoo, Finland

*Corresponding author: email: juha.rouvinen@uef.fi

Contents:

Figure S1. FID chromatograms of the anisole oxyfunctionalisation products by UPO M6.

Figure S2. FID chromatograms of the anisole oxyfunctionalisation products by *Aae*UPO.

Figure S3. FID chromatograms of the anisole oxyfunctionalisation products by UPO 6.

Figure S4. Mass spectra of the identified compounds in anisole oxyfunctionalisation reactions.

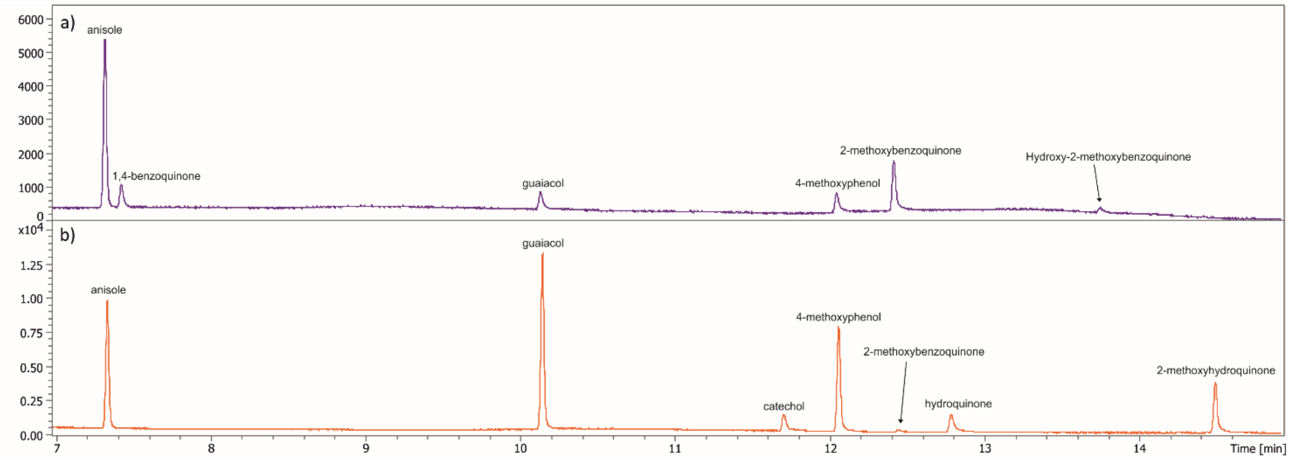

Figure S1. FID chromatograms of anisole oxyfunctionalisation products by UPO M6. The reactions were performed a) without and b) with ascorbic acid. 3-Methoxycatechol, 4-methoxyresorcinol and 4-methoxycatechol products were observed only by EICs.

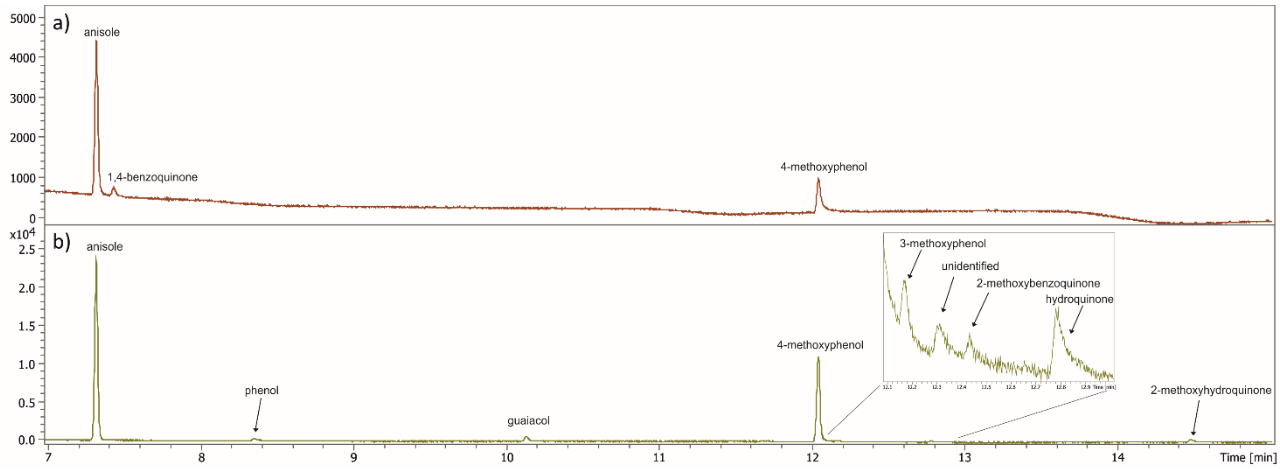

Figure S2. FID chromatograms of anisole oxyfunctionalisation products by *Aae*UPO. The reactions were performed a) without and b) with ascorbic acid. The inset in shows a magnified view at the retention time frame of 12.1 to 12.9 min.

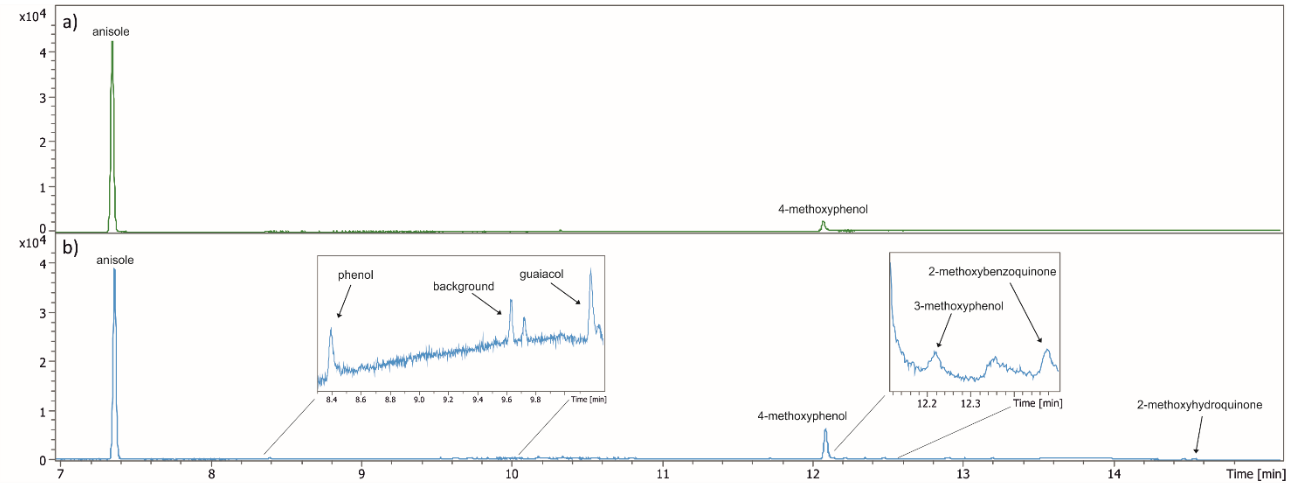

Figure S3. FID chromatograms of anisole oxyfunctionalisation products by UPO 6. The reactions were performed a) without and b) with ascorbic acid. The insets in b) show magnified views at the retention time frames of 8.3 to 10.2 min and 12.1 to 12.5 min.

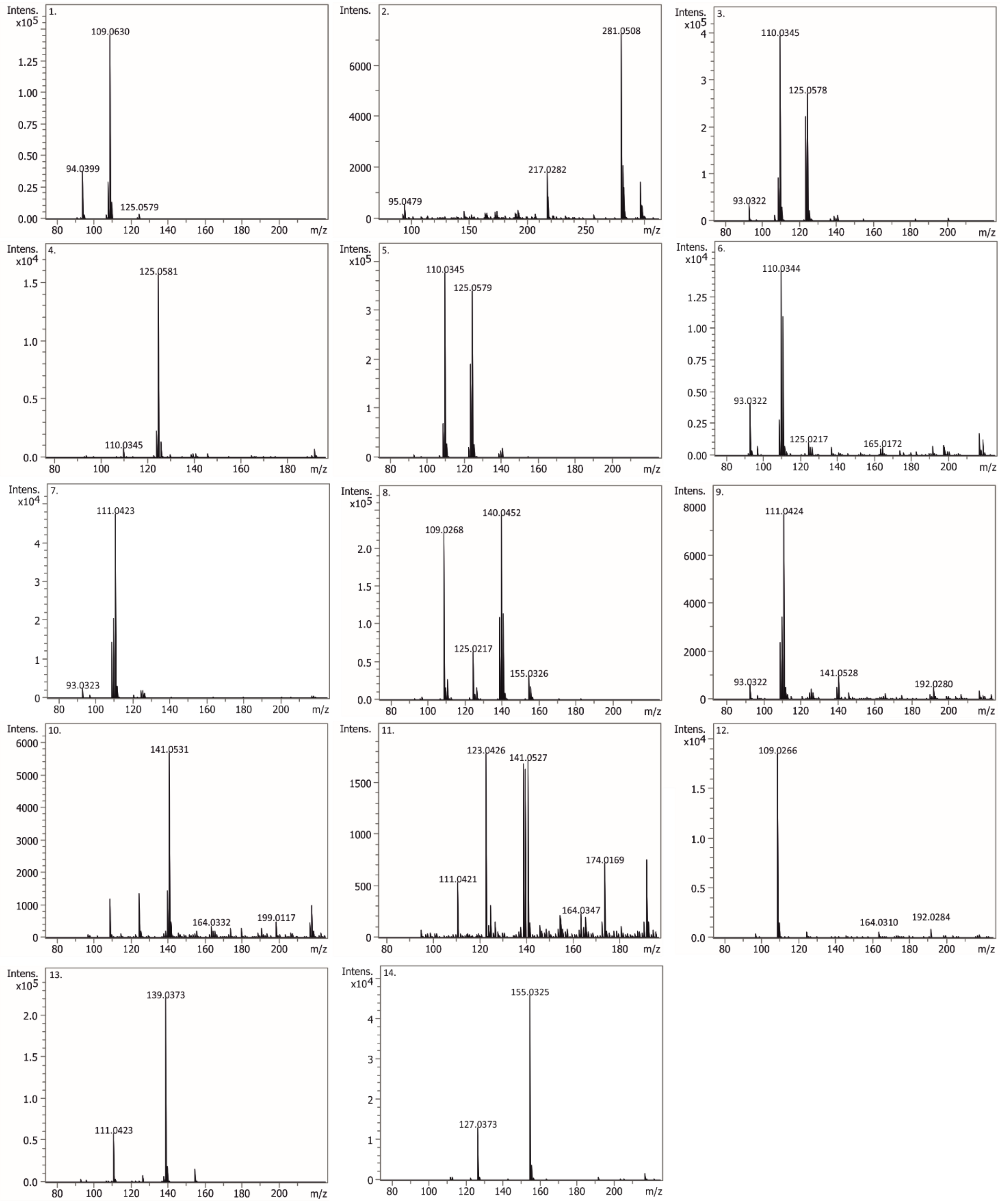

Figure S4. Mass spectra of the compounds identified in the anisole oxyfunctionalisation reactions: 1) anisole (substrate), 2) phenol, 3) guaiacol, 4) 3-methoxyphenol, 5) 4-methoxyphenol, 6) catechol, 7) hydroquinone, 8) 2-methoxyhydroquinone, 9) 3-methoxycatechol, 10) 4-methoxyresorcinol, 11) 4-methoxycatechol, 12) 1,4-benzoquinone, 13) 2-methoxybenzoquinone, and 14) hydroxy-2-methoxybenzoquinone.
